# The first case of intellectual disability caused by novel compound heterozygosity for *NUDT2* variants

**DOI:** 10.1186/s12887-024-04542-3

**Published:** 2024-01-19

**Authors:** Bo Bi, Xiaohong Chen, Shan Huang, Min Peng, Weiyue Gu, Hongmin Zhu, Yangcan Ming

**Affiliations:** 1 Department of Rehabilitation, Wuhan Children’s Hospital, Tongji Medical Colllege, Huazhong University of Science and Technology, Wuhan, Hubei China; 2 School of Medicine, Wuhan University of Science and Technology, Wuhan, Hubei China; 3 Chigene (Beijing) Translational Medical Research Center Co., Ltd, Beijing, China; 4 Department of Pediatrics, Traditional Chinese and Western Medicine Hospital of Wuhan, Tongji Medical College, Huazhong University of Science and Technology, Wuhan No. 1 Hospital, Wuhan, Hubei China; 5 College of Chinese Medicine, Hubei University of Chinese Medicine, Wuhan, Hubei China

**Keywords:** Developmental delay, Intellectual disability, Motor delay, *NUDT2*

## Abstract

NUDT2 is an enzyme important for maintaining the intracellular level of the diadenosine tetraphosphate (Ap4A). Bi-allelic loss of function variants in *NUDT2* has recently been reported as a rare cause of intellectual disability (ID). Herein, we describe a Chinese girl with ID, attention deficit hyperactivity disorder (ADHD), and motor delays with abnormal walking posture and difficulty climbing stairs, who bears compound heterozygous variants c.34 C > T (p.R12*) and c.194T > G (p.I65R) in *NUDT2*. Homozygous variants c.34 C > T (p.R12*) or c.186del (p.A63Qfs*3) in *NUDT2* were previously reported to cause ID. This is the first patient with ID due to compound heterozygous variants in *NUDT2* and p.I65R is a novel missense variant. This study enriched the genotype and phenotype of *NUDT2-*related ID and supported the critical developmental involvement of *NUDT2.*

## Introduction

Global developmental delay (GDD) and intellectual disability (ID) are phenotypically and genetically heterogeneous with common morbidity affecting at least 2 ~ 3% of the population [1]. Investigation for genetic causes of GDD/ID has contributed to the identification of hundreds of disease-causing genes. However, challenges remain in the specific diagnosis, as there is significant variability in the clinical features. Trio-based whole-exome sequencing (Trio-WES) analysis gives a high diagnostic yield of over 50% in children with severe developmental delay if used as a first-tier diagnostic test [2]. NUDT2 (Nudix hydroxylase 2; Nudix-type motif 2) is a member of the MutT family of nucleotide pyrophosphatases, which catalyzes the asymmetric hydrolysis of Ap4A to yield AMP and ATP. NUDT2 also exhibits decapping activity towards FAD or dpCoA-capped RNAs in vitro and involves viral RNA degradation and antiviral immunity [3, 4]. This gene may be a prognostic marker in breast carcinomas [5–7]. So far, 11 GDD/ID patients have been reported exhibiting homozygous variants in *NUDT2*, suggesting a neurodevelopmental role [8–11]. Here, we performed Trio-WES and identified novel compound heterozygosity for *NUDT2* variants in a Chinese patient, affected with GDD/ID, but also with ADHD, further expanding the genotypic and phenotypic spectrum associated with this gene.

## Materials and methods

### Whole-exome sequencing

Trio-WES and Sanger sequencing was performed on genomic DNA extracted from the peripheral blood from the proband and her parents. Libraries were prepared using xGen Exome Research Panel v1.0 (IDT, Iowa, USA) followed by sequencing on the Illumina NovaSeq 6000 (Illumina San Diego, CA). Sequences underwent processing, including adapter trimming and filtering of low-quality reads using fastp, alignment to the GRCh37/hg19 reference genome was performed with Burrows-Wheeler Aligner (BWA), and variant calling of single nucleotide variants (SNVs) as well as insertions and deletions < 50 bp (small Indels) was executed by Genome Analysis Tool Kit (GATK) [12–14]. Basic filtering excluded the following variants: (1) Wild-type in the proband, (2) Intronic variants deeper than 30 bp, (3) Low-quality SNPs (variant frequency < 0.2 or sequencing depth < 4X or quality value < 35), (4) Low-frequency simple tandem repeat Indels (SSR > 7 and AF < 0.3), (5) Indels larger than 50 bp. Annotation of high-quality variants utilized dbSNP, gnomAD, 1000 Genomes Project, ExAC, ESP, and Chigene in-house MAFs database. Additional databases such as OMIM, HGMD, and ClinVar were used. Annotation was performed using a variant annotation software independently developed by Beijing Chigene Translational Medicine Research Center Co., Ltd, 100,875, Beijing. Computational prediction tools, including Provean, SIFT, Polypen2, MutationTaster, M-Cap, and REVEL software packages, were employed to assess the potential pathogenicity of missense variants. MaxEntScan, dbscSNV, and GTAG software packages were used to predict the functional impact of variants on splicing sites. Variant prioritization focused on rare variants with moderate or high predicted impacts, such as frameshift, nonsense, splicing, and missense. Phenotype consistency was evaluated through Human Phenotype Ontology (HPO) terms. Trio analysis assessed de novo, compound heterozygous, and recessive models. Top candidate variants underwent manual review, considering IGV visualization, inheritance patterns, gene function, and patient phenotype. WES identified potential pathogenic variants aligned with the patient’s phenotype and family history. All variants were assessed following the American College of Medical Genetics and Genomics (ACMG) guidelines [15]. The identified variants and segregation analysis were confirmed by Sanger sequencing using specific primers.

## Results

### Clinical description

**Fig. 1:**
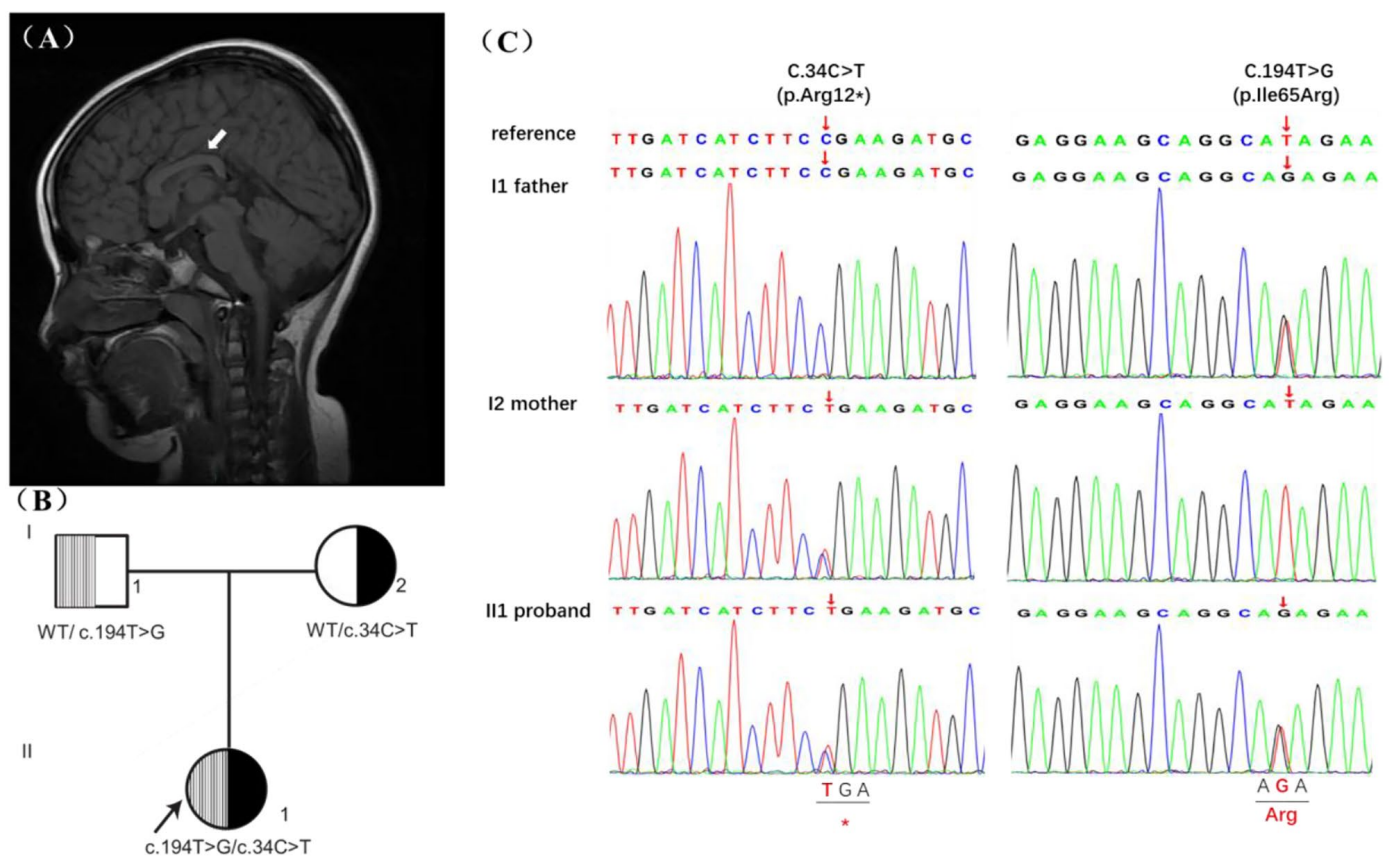
A Chinese family with *NUDT2* compound heterozygous variants causing intellectual disability. **(A)** Sagittal brain MRI of the patient. Note thinning of the corpus callosum (arrow). **(B)** Pedigree of the family. An arrow indicates the proband. The patient (II-1) had compound heterozygous variants p.I65R and p.R12*, which were inherited from her father (I-1) and mother (I-2), respectively. **(C)** Sanger sequencing pherograms show compound heterozygosity of *NUDT2* in the proband and heterozygosity in both parents

The proband was a 5-year-old female to healthy non-consanguineous parents of Chinese ethnicity. She was born at week 40^+ 5^ by caesarian section with a birth weight of over 3 kg and a length of 52 cm. No history of neonatal complications or feeding difficulties was observed. Milestones of motor development were delayed as she sat at 9 months, stood at 2 years, and walked unsupported at 2 years 4 months. For her speech, she vocalized “mama, baba” at 10 months and began using single words at 1 year. On current examination at 6 years, her growth parameters weight (30 kg, 97th centile), and height (123 cm, 90th centile) were within normal range. She had subtle facial dysmorphism, such as slightly upslanting palpebral fissures. Her language expression lagged behind that of other children of the same age and slightly poor logic of speech with unclear articulation. Testing with the Wechsler Intelligence Scale revealed 84 for the verbal IQ, 63 for the performance IQ, 71 for the full-scale IQ, showing cognitive skills mildly impaired. She presented with ADHD and deficits in executive function, including slow processing speed, poor learning, and working memory performance. Muscular hypotonia in all extremities and motor impairments with an unsteady gait, climbing stairs difficulties were also noted. Brain magnetic resonance imaging (MRI) revealed thinning but fully formed corpus callosum **(**Fig. 1A**)**, without other abnormalities. Electroencephalogram (EEG) was normal. Electromyography (EMG) examination demonstrated sensorimotor neurogenic lesions in the distal lower extremities with axonal features in a length-dependent manner, and a normal nerve conduction velocity and SNAP amplitudes.

### Molecular analysis

**Fig. 2:**
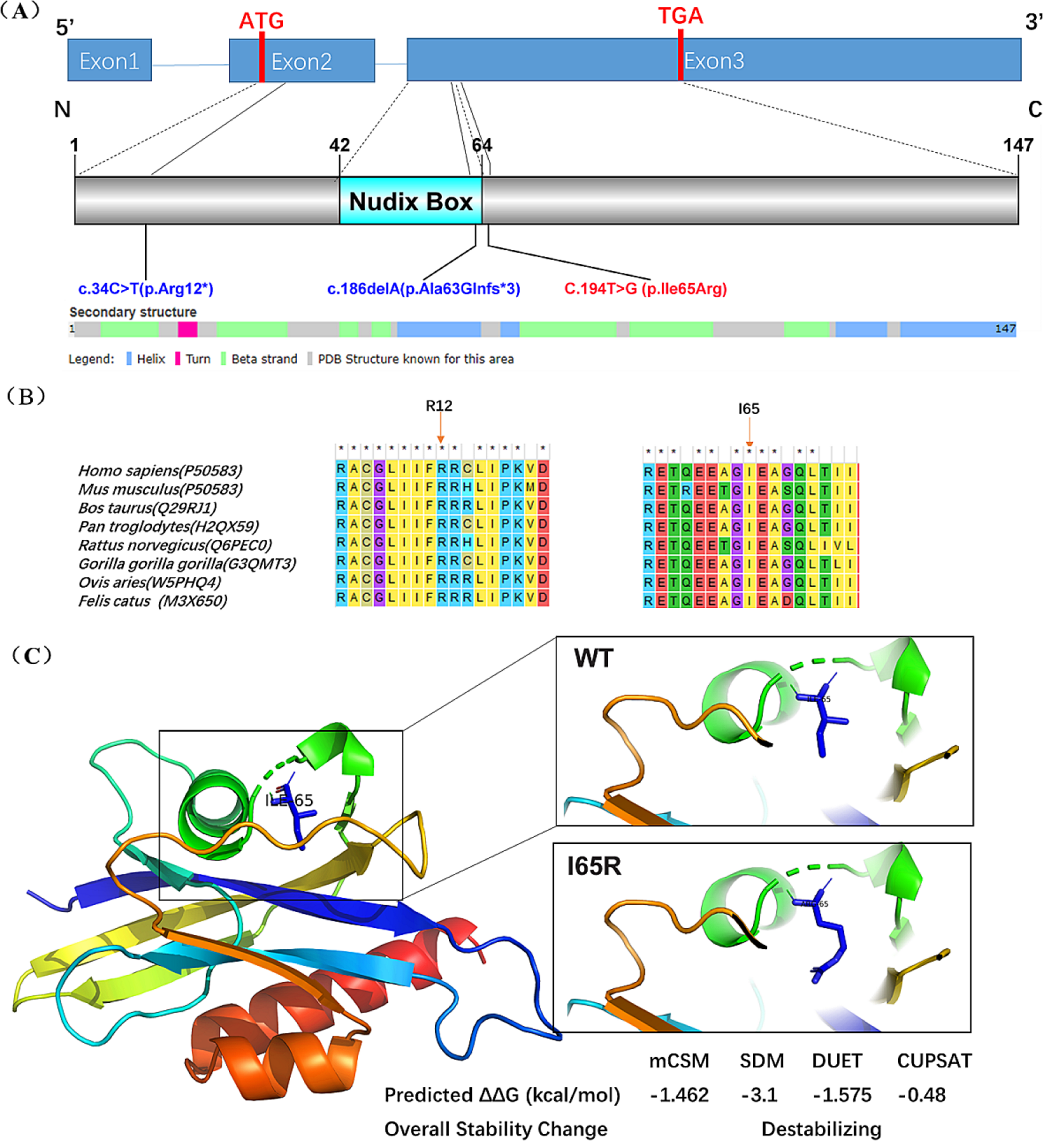
Identification of a novel *NUDT2* missense variant. **(A)** Schematic representation of *NUDT2* transcript (NM_001244390) and protein (NP_001231319) with the light blue box delineates the catalytically active Nudix Box. Previously identified variants in GDD/ID are indicated in blue. The identified novel variant is indicated in red. The secondary structure of NUDT2 protein (six beta-strands and four helixes) shows I65 located at the loop between the first and second helixes. **(B)** Conservation of the identified variants in *NUDT2*. The residue R12 and I65 (marked with red arrows) are highly conserved among species. **(C)** Location of I65R mutant in NUDT2 protein (AlphaFold: AF-P50583-F1) and the fold stability change prediction (ΔΔG) upon I65R mutant using four methods (mCSM/SDM/DUET/CUPSAT). I65R destabilizes overall stability

Trio-WES revealed the proband harboring compound heterozygous variants at *NUDT2* NM_001244390: c.34 C > T (p.R12*) and c.194T > G (p.I65R), which were inherited from his mother and father, respectively **(**Fig. 1B**)**. Sanger sequencing further confirmed both asymptomatic parents to be heterozygous carriers, following the rule of autosomal recessive inheritance **(**Fig. 1C**)**. Following the ACMG guidelines, the c.34 C > T (NM_001244390) variant in exon 2 is classified as pathogenic, documented in dbSNP with rs number rs148119952. The variant has a frequency of 0.0024 in the 1000 Genomes Project and is absent in gnomAD. It is a nonsense mutation (PVS1), expected to result in premature protein termination. The pathogenic p.R12* has been reported as a homozygous LOF variant in seven GDD/ID patients (PM3) and showed genotype-phenotype segregation in one family (PP1-Moderate). The missense variant of uncertain significance (VUS) p.I65R is absent in the population databases such as 1000 genomes, ExAC, ESP and gnomAD or our internal database of exomes. So it is considered a rare variant although this variant has been recorded in the dbSNP database with the rs number rs765777597 (PM2). Various protein damage prediction software tools, including MutationTaster and SIFT, suggest that this variant is deleterious (PP3). Of the six missense variants in ClinVar, c.174G > T (p.Glu58Asp) was likely pathogenic in intellectual developmental disorder with or without peripheral neuropathy (autosomal recessive inheritance), submitted by the University Hospital Tübingen. Furthermore, residue I65 is positioned near the Nudix box domain of NUDT2 and is highly evolutionary conserved across species **(**Fig. 2A and B**)**. In molecular modeling, I65 localizes in the loop between the first and second helixes, if it mutates to R65, which replaces hydrophobic isoleucine with a basic polar arginine, will generate unfavorable torsion angles and lead to a change in protein thermodynamic stability (ΔΔG) and thus destabilize the structure of NUDT2, indicating a likely impact on the protein function **(**Fig. 2C**)**. No other rare variants were identified by Trio-WES in the patient that could explain the observed phenotype. With her clinical profile, especially the presence of ID and delayed motor development resembling the reported cases’ clinical presentations, we considered the identified *NUDT2* variants as the likely cause explaining our patients’ disease phenotypes.

## Discussion

*NUDT2*, located at position 9p13.3, contains 3 exons and encodes a 147-amino acid Ap4A hydrolase containing a MutT motif or“nudix”. The protein is believed to play a major role in maintaining the low level of intracellular Ap4A, the function of which has yet to be established [6]. *NUDT2-*related ID is a rare entity. The variants identified in *NUDT2* so far included one nonsense and one frameshift variant that exert their effect through loss-of-function (LOF) [8–11]. Thus, our knowledge regarding *NUDT2* defects that lead to clinical manifestations of GDD/ID is limited. The present study describes a 5 years-old female from a Chinese family with ID, who bears one known nonsense variant and a newly described missense variant, supporting the previous findings and expanding the mutational spectrum for autosomal recessive ID.

Until now, only 11 reported cases with neurological symptoms due to two variants in the *NUDT2* gene have been described in 7 pedigrees in homozygous conditions (Table 1). Most cases were in consanguine families of Saudi Arabian origin (7 cases, 64%), two cases were of Mexican descent and one case was of Cajun descent. *NUDT2*-related disorders included ID and GDD. Additional signs comprised hypotonia, delayed motor and language development, and cognitive impairment; and less frequently, ataxia, leukodystrophy, thinning of the corpus callosum, denervation atrophy, demyelination, or axonal sensorimotor polyneuropathy. Non-neurological features encompassed low birth weight and height, neonatal feeding problems, subtle facial dysmorphism, and microcephaly. The first and second studies described seven cases with homozygous p.R12* in *NUDT2* to underlie ID/GDD [8, 9]. The four patients presented in the third and fourth studies with homozygous p.A63Qfs*3 in *NUDT2* also developed polyneuropathy in addition to ID [10, 11]. We report the first case cosegregated with compound heterozygous mutations (p.R12* and p.I65R) in *NUDT2*. Although our case presented some of the recognized features, ADHD is first described here, and low birth weight and height, weak sucking in infancy, and microcephaly were not observed in our patient. Our case also showed a length-dependent axonal sensorimotor polyneuropathy, which suggested that progressive sensorimotor neuropathy may be invariably present. The two affected sisters with homozygous p.R12*, walked by 4 years of age and vocalized “mama, baba” at 2.5 years [8]. The three patients with homozygous p.A63Qfs*3 walked at the age of 3 and began using single words at age 2 [10]. In contrast, our patient walked unsupported at 2 years 4 months. Her parents noticed no delays in language development but recently noted a decrease in speech articulation. Truncating variant p.R12* at N-terminal is predicted to trigger nonsense-mediated decay (NMD) and impair the enzymatic domain and p.A63Qfs*3 in the last exon is unlikely to undergo NMD. The missense mutation p.I65R reported here resides in a conserved region near the Nudix box domain but may not define pathogenic LOF alleles, thereby differential activities of the protein placing *NUDT2*-related disease towards the milder phenotypes of neurodevelopmental disorders.

**Table 1:** Biallelic *NUDT2* variants in 12 individuals and associated phenotypes

Fam	Case	Gender	Age	Zygosity	Allele1	Allele2	GDD	ID	HT	Impaired speech	Motor delay	MC	Facial dysmorphism	Brain abnormality on MRI	Neuropathy	References
A	1	Female	10y	Hom	Nonsense; c.34 C > T; p.Arg12*	Nonsense; c.34 C > T; p.Arg12*	+	+	+	+	+	ND	+	+	ND	Anazi et al.
A	2	Female	7y	Hom	Nonsense; c.34 C > T; p.Arg12*	Nonsense; c.34 C > T; p.Arg12*	+	+	+	+	+	ND	+	+	ND	Anazi et al.
B	3	Female	ND	Hom	Nonsense; c.34 C > T; p.Arg12*	Nonsense; c.34 C > T; p.Arg12*	+	+	+	+	+	+	+	+	ND	Yavuz et al.
B	4	Male	ND	Hom	Nonsense; c.34 C > T; p.Arg12*	Nonsense; c.34 C > T; p.Arg12*	+	+	+	+	+	+	+	+	ND	Yavuz et al.
B	5	Male	ND	Hom	Nonsense; c.34 C > T; p.Arg12*	Nonsense; c.34 C > T; p.Arg12*	+	+	+	+	+	+	+	+	ND	Yavuz et al.
C	6	Male	ND	Hom	Nonsense; c.34 C > T; p.Arg12*	Nonsense; c.34 C > T; p.Arg12*	+	+	+	+	+	+	+	+	ND	Yavuz et al.
D	7	Female	ND	Hom	Nonsense; c.34 C > T; p.Arg12*	Nonsense; c.34 C > T; p.Arg12*	+	+	+	+	+	+	+	+	ND	Yavuz et al.
E	8	Male	23y	Hom	Frameshift; c.186del; p.Ala63Glnfs*3	Frameshift; c.186del; p.Ala63Glnfs*3	+	+	ND	+	+		+		+	Diaz et al.
E	9	Female	10y	Hom	Frameshift; c.186del; p.Ala63Glnfs*3	Frameshift; c.186del; p.Ala63Glnfs*3	+	ND	ND	+	+		+	ND	+	Diaz et al.
F	10	Female	12y	Hom	Frameshift; c.186del; p.Ala63Glnfs*3	Frameshift; c.186del; p.Ala63Glnfs*3	+	ND	ND	+	+		+		+	Diaz et al.
G	11	ND	21y	Hom	Frameshift; c.186del; p.Ala63Glnfs*3	Frameshift; c.186del; p.Ala63Glnfs*3	+	+	ND	ND	+	ND	+	+	+	Maddirevula et al.
H	12	Female	5y	Com het	Nonsense; c.34 C > T; p.Arg12*	Missense; c. 194T > G; p. Ile65Arg	+	+	+	+	+		+	+	+	This report

Abbreviations: Fam, family identification; ND, not mentioned; y, years; Com het, compound heterozygous; Hom, homozygous; GDD, global developmental delay; ID, intellectual disability; HT, hypotonia; MC, microcephaly; MRI, magnetic resonance imaging. All the variants are described using the NM_001244390 (GRCh37/hg19) transcript and NP_001231319 protein of *NUDT2*.

In conclusion, this study contributed further to the characterization of *NUDT2*-related disorders and identified novel compound heterozygosity as the cause of disease, allowing for accurate genetic counseling. Our results supported that variants in the *NUDT2* cause a multisystem disease with intellectual disability and polyneuropathy, and more research is needed to study the underlying mechanisms of *NUDT2*-related disorders and the genotype-phenotype correlations. The clinical possibility of *NUDT2* biallelic mutation should be considered in children with GDD/ID.

## Data Availability

All data generated or analysed during this study are included in this published article. The original contributions presented in this study are publicly available. The *NUDT2* variants NM_001161.4: c.34 C > T/p.Arg12*, c.194T > G/p.Ile65Arg were submitted to the LOVD database (https://databases.lovd.nl/shared/transcripts/NUDT2), with the LOVD Variant ID: https://databases.lovd.nl/shared/variants/0000932973#00014895, https://databases.lovd.nl/shared/variants/0000932972#00014895.
